# Pain experience and perception in individuals with Snijders Blok-Campeau syndrome

**DOI:** 10.3389/fpain.2025.1540422

**Published:** 2025-08-13

**Authors:** Don Daniel Ocay, Philippe M. Campeau, Charles B. Berde, Catherine A. Brownstein

**Affiliations:** ^1^ Department of Anesthesiology, Critical Care and Pain Medicine, Boston Children’s Hospital, Boston, MA, United States; ^2^ Department of Anaesthesia, Harvard Medical School, Boston, MA, United States; ^3^ Department of Pediatrics, Université de Montréal, Montreal, QC, Canada; ^4^ The Manton Center for Orphan Disease Research, Boston Children’s Hospital, Boston, MA, United States; ^5^ Division of Genetics and Genomics, Boston Children’s Hospital, Boston, MA, United States; ^6^ Department of Pediatrics, Harvard Medical School, Boston, MA, United States

**Keywords:** pain, experience, perception, Snijders Blok-Campeau syndrome, *CHD3*

## Abstract

**Introduction:**

Snijders Blok-Campeau Syndrome (SNIBCPS) is a neurodevelopmental disorder characterized by intellectual disability, developmental delays, speech impairment, hypotonia, and distinctive facial features. Little is known about pain perception in children with cognitive impairments, such as patients with SNIBCPS. Although it has been noted that some individuals with SNIBCPS have decreased pain sensation and response to painful stimuli, these reports are anecdotal. Therefore, the objective was to better understand this syndrome and the affected individual's perception and response to pain through proxy-reported observational assessments.

**Methods:**

Fifteen caregivers of individuals with a diagnosis of SNIBCPS participated in this mixed-methods anonymous survey study between July and September 2024. The survey questionnaires included the Pediatric Pain Profile, a Pain Sensory Questionnaire, the Non-Communicative Children's Pain Checklist-Revised, and the Individualized Numerical Rating Scale.

**Results:**

Almost a quarter of our respondents reported insensitivity in the affected individual to hard impacts or pressure. Our findings highlight early and past painful experiences in individuals with SNIBCPS who have a range of behaviors to express their pain.

**Discussion:**

Our findings bring awareness about the proper examination of individuals with SNIBCPS. Despite the small sample size, our findings suggest that pain and injuries may go unreported in individuals with SNIBCPS, and individualized parental observational scales may be beneficial for their healthcare providers and their caregivers.

## Introduction

Snijders Blok-Campeau Syndrome (SNIBCPS, OMIM# 618205) is a rare neurodevelopmental disorder caused by mutations in the Chromodomain Helicase DNA Binding Protein 3 (*CHD3*) gene (1). The *CHD3* gene is located on chromosome 17p13.1, and encodes a protein that is part of a chromatin remodeling complex called NuRD and plays an important role in regulating gene expression during early brain development. Mutations in *CHD3* disrupt this process, leading to dysregulation of gene expression during critical stages of brain development.

SNIBCPS is characterized by intellectual disability, developmental delays, speech impairment, hypotonia, and distinctive facial features (1, 2). The degree of intellectual disability ranges from mild to severe, with most cases being moderate to severe (1–3). Other associated features may include seizures, autism spectrum disorder, behavioral problems, macrocephaly or more rarely microcephaly, and congenital malformations. The disorder follows an autosomal dominant inheritance pattern, meaning a single mutated copy of the *CHD3* gene is sufficient to cause the condition. Various types of mutations in the *CHD3* gene have been identified, including nonsense, missense, frameshift, and splice site mutations (1–7). Since there is no specific treatment for *CHD3*-related intellectual disability, management is primarily supportive and involves early intervention with speech, physical, and occupational therapies, as well as educational support and management of associated medical issues.

There is limited research about pain perception in children with cognitive impairments, such as patients with SNIBCPS. Importantly, children with severe cognitive impairments may express pain differently due to difficulties with communication, and are unable to provide self-reported pain intensities (8, 9). This has led to the assumption that children with cognitive impairments have decreased pain sensitivity (10). However, studies have shown in individuals with cognitive impairment, such as those with autism spectrum disorder, conflicting results, with one study reporting no difference in sensory function when compared to matched healthy controls (11, 12). Although it has been noted that four individuals with SNIBCPS have decreased pain sensation and response to painful stimuli, these reports are anecdotal (1, 2). Moreover, the absence of pain expression does not represent the absence of pain perception. Pain that is not managed properly could significantly decrease quality of life (13). Therefore, observational assessments of pain are used to better understand how patients with cognitive impairments, such as SNIBCPS, perceive pain. Nevertheless, empathy and compassion need to be prioritized in this population to foster a safe environment where the individual can express discomfort through non-verbal responses (14).

To our current knowledge, there are limited data on the pain perception of individuals with SNIBCPS. Therefore, the objective was to better understand this syndrome and their perception and response to pain through proxy-reported observational assessments. Our aims were to (1) determine the pain history and (2) determine the expression of pain of individuals with SNIBCPS. We hypothesized that individuals with SNIBCPS may experience additional sources of pain compared to typically developing children, and display different behaviors during a painful situation compared to a non-painful situation.

## Materials and methods

### Study approval, participants, and experimental design

Ethics approval was obtained from the Research Ethics Board of our Institution (IRB# P00045527). Caregivers (parents/legal guardians) of individuals with a diagnosis of SNIBCPS above the age of 18 years old were offered to participate in this mixed-methods survey study. Through a collaboration with the CHD3 Foundation (https://www.chd3.org/), targeted emails were sent once a month to their members between July and September 2024 to inform potential families to participate anonymously in this study. Because this was an anonymous survey, no protected health information was collected, and consent was provided by the completion of the survey. No incentive or compensation was offered to participants who completed the survey.

### Outcome measures

The primary outcome variables included the questionnaires below. Secondary outcome measures include demographic data such as the current age, gender, and race of the respondent, as well as the current age, age of diagnosis, gender, and race of the child.

• The Pediatric Pain Profile (PPP) is an observational tool developed to assess and monitor pain in children with severe cognitive impairments and unable to verbally express their pain (8). The PPP is a validated 20 item behavior rating scale designed to interpret behaviors or signs of pain (15, 16). Each item is rated on a four-point Likert scale from “not at all” to “a great deal”. Caregivers were asked to assess the pain profile of the children's behaviour when they are “on a good day” and when they experience their “most troublesome pain” if applicable. Scores range from 0 to 60 in which scores of 14 or more are generally associated with moderate or severe pain. The pain history of the children was also collected to know how the child has coped with pain and injury in the past.
• The Pain Sensory questionnaire (PSQ) is an observational tool developed to understand what triggers a child's pain and what does not. The questionnaire has previously been used in a cohort of caregivers of individuals with Christianson syndrome (17). The first section of the questionnaire asks whether the child is insensitive or sensitive when faced different sensations (cold, heat, light touch, pressure, hard impact, gusts of air, smooth surface, rough surface). If the child was sensitive to a specific sensation, a follow-up question was asked to determine whether the child has an aversive reaction.
• The Non-Communicative Children's Pain Checklist—Revised (NCCPC-R) is an observational tool designed to describe the behaviors of a child with cognitive impairment or disabilities over a period of 2 h in a home setting (18). The NCCPC-R is a validated 30-item behavior frequency rating scale divided into seven sub-scales (vocal, social, facial, activity, body and limbs, physiological, and eating/sleeping) designed to describe the child's behavior when in pain. Each item is rated on a five-point Likert scale (0 = not at all, 1 = just a little, 2 = fairly often, 3 = very often, NA = not applicable). The caregivers were asked to fill out the NCCPC-R to describe the child's reactions or lack of reaction to different painful situations if applicable. The total scores range from 0 to 90 in which a score of 7 or more indicated that the child is experiencing pain.
• The Individualized Numeric Rating Scale (INRS) is a 0–10 numerical rating scale that includes space for caregivers to insert typical pain responses for a nonverbal individual with cognitive impairment (19, 20). Building upon the NRS in which numbers ranging from 0 to 10 are placed at equidistant points on a line where 0 equals no pain and 10 equals the worst pain imaginable), caregivers were asked to populate patient pain behaviors on the vertical line that corresponds to pain intensity for their child.

### Sample size and statistical analysis

Given the rarity of this syndrome, there was no sample size calculated. However, with approximately 170 known cases, according the CHD3 Foundation's website, and approximately 3%–5% of those emailed are expected to digitally consent and respond to at least one survey question, a desired sample of at least 5 was envisioned.

Initial descriptive analyses of all demographic variables was conducted by calculating the means or medians and standard deviations or ranges for the continuous outcomes, and cross-tabulations for the categorical measures. For the first aim, summative qualitative content analysis was conducted to summarize the pain history of all children of the respondents, and how their child has coped with pain and injury/illness in the past. Summative qualitative content analysis which consists of interpreting, classifying, and comparing the comments to determine themes and patterns (21). One of the authors (DDO) independently read the transcripts multiple times to identify categories of similar comments. For the secondary aim, descriptive analyses of all questionnaires was conducted. Moreover, summative qualitative content analysis of the responses from the INRS was conducted to determine any themes used for caregivers descriptions of pain intensities.

## Results

The survey was viewed 35 times and was started by 30 people. Among those, 25 respondents completed the demographics questionnaire, 15 respondents completed the first questionnaire (PPP), and six respondents completed the final questionnaire (INRS) leading to a 20% completion rate. The demographics of the 15 respondents/caregivers who completed the first questionnaire is summarized in Table 1. Five (33%) of the children were toddlers (1–3 years), five (33%) were of pre-school age (3–6 years), three (20%) were school-age (7–12 years), and two (13%) were adolescents (13–18 years).

**Table 1:** Demographic characteristics of sample, *N* = 15.

Demographic variable	Respondent	Child
Age (years), median [range]	39 [32–54]	5 [1–15]
Gender		
Male	5 (33)	11 (73)
Female	10 (67)	3 (20)
Prefer not to say	0	1 (7)
Ethnicity		
Indigenous, American Indian or Alaska Native	0	0
Asian	0	0
Black or African American	0	0
Hispanic or Latino	2 (13)	2 (13)
Native Hawaiian or Other Pacific Islander	0	0
White	13 (87)	12 (80)
Interracial	0	1 (7)
Prefer not to say	0	0
Age at diagnosis (years), median [range]		3 [1–13]

Data presented as *N* (%) unless otherwise specified.

### Pain history of individuals with SNIBCPS

Fifteen respondents completed the pain history section of the Pediatric Pain Profile, while only thirteen (87%) completed the baseline assessments (Table 2). Only eight respondents (53%) reported early/past pain experiences from their child, such as being an inpatient in the neonatal intensive care unit, infections, illnesses, needles and injuries. However, only three of those respondents (38%) reported observing “normal” pain behaviors, such as crying or seeking comfort. Only 7 respondents (47%) reported past surgical experience in their child. Four of those respondents (57%) did not report or observe pain behaviors after surgery from their child. Eight respondents (53%) reported illnesses and injuries, such as ear infections, bumps, falls, and colds. However, there was an even split by the respondents in reporting tolerable behavior, or a normal or heightened response to the illness or injury.

**Table 2:** Responses for the pediatric pain profile, *N* = 15.

Early/past pain experiences, *N* = 15	*N* (%)
As an infant	• Did not report any early/past experiences = 3 (20) • Unable to identify pain/distress = 3 (20) • Reported early/past pain experiences (e.g., NICU, infection/illness, needles, injury) = 8 (53) ○ Observed “reduced” pain behaviors = 4 (27) ○ Observed “normal” pain behaviors = 3 (20)
Surgery	• Did not report/deny any past surgery = 4 (27) • Reported no past surgery = 4 (27) • Reported surgery = 7 (47) ○ Testicular = 2 (13) ○ Eye = 1 (7) ○ Ear = 2 (13) ○ Other/Not specified = 4 (27) • Did not report/observe pain after surgery = 4 (27) • Reported increased irritability = 1 (7)
Illness and injury	• Did not report/deny any illness/injury = 5 (33) • Ear infections = 3 (20) • Bumps, falls = 3 (20) • Stomach aches, colds = 2 (13) • Tolerable to illnesses/injuries reported = 4 (27) • Normal/heightened responses reported = 4 (27)
On a good day, *N* = 13	
Total score, median [range]	7 [2–24]
Moderate to severe pain (Total score ≥ 14)	4 (31)
Pain even on a good day like this?	
No pain	10 (77)
Mild pain	3 (23)
Moderate to very severe pain	0
Any current or recurring pains?	3 (23)
Participant 12, total score = 21, Described as “Mild pain”	Stomach constipation; Present throughout whole life (every day), but primarily during the night time. Medication alleviates pain
Participant 16, total score = 45, Described as “Very severe pain”	Ankle pain due to hypermobility and multiple sprains; Lasts around 4 months due to physical activity (jumping, running). Alleviated with ice
Participant 16, total score = 50, Described as “Very severe pain”	Experiences pain while brushing teeth, even when using soft bristles; Occurred upon commencing brushing teeth as a child. The child is very reluctant to brush teeth and adds that they do not like the texture, taste and smell of toothpaste. Nothing alleviates the pain
Participant 27, Total score = 10, Described as “Mild pain”	Pain in the ears; Occurs when child has a cold, lasting 6 months or more, throughout the day. Tylenol alleviates the pain

Data presented as *N* (%) unless otherwise specified.

From the baseline assessments of the respondents, even on a good day, three (23%) respondents reported mild pain in their child. Three respondents reported recurring pain in their child, with two of those respondents reporting their child is experiencing “mild pain”, while the other respondent reported their child was experiencing “very severe pain”.

### Pain expression of individuals with SNIBCPS

Thirteen respondents completed the Pain Sensory Questionnaire (Table 3). Up to three respondents (23%) reported their child to be insensitive to specific sensations, particularly pertaining to deep pressure and hard impact. On the other hand, two to five of the respondents (15%–38%) reported their children being sensitive to most sensations, with at least 50% of these respondents reporting that these sensations would trigger an aversive reaction in their children. For example, four of the respondents (31%) reported their children being sensitive to innocuous stimuli such as gusts of air, with all of these respondents reporting an aversive reaction from their children. Our findings highlight a wide range of sensitivity responses to noxious and innocuous stimuli.

**Table 3:** Responses for the pain sensory questionnaire, *N* = 13.

Sensation	Sensitivity			
	Insensitive	Normal	Sensitive	Aversive reaction
Cold	1 (8)	7 (54)	5 (38)	4 (80)
Heat	0	11 (85)	2 (15)	1 (50)
Light touch	0	10 (77)	3 (23)	2 (67)
Pressure	3 (23)	8 (62)	2 (15)	2 (100)
Hard impact	3 (23)	8 (62)	2 (15)	1 (50)
Gusts of air	0	9 (69)	4 (31)	4 (100)
Smooth surface (e.g., stroking on skin or glass surface)	0	11 (85)	2 (15)	1 (50)
Rough surface (e.g., sandpaper or rocks)	0	9 (69)	4 (31)	3 (75)

Data presented as *N* (%) unless otherwise specified.

**Figure 1:**
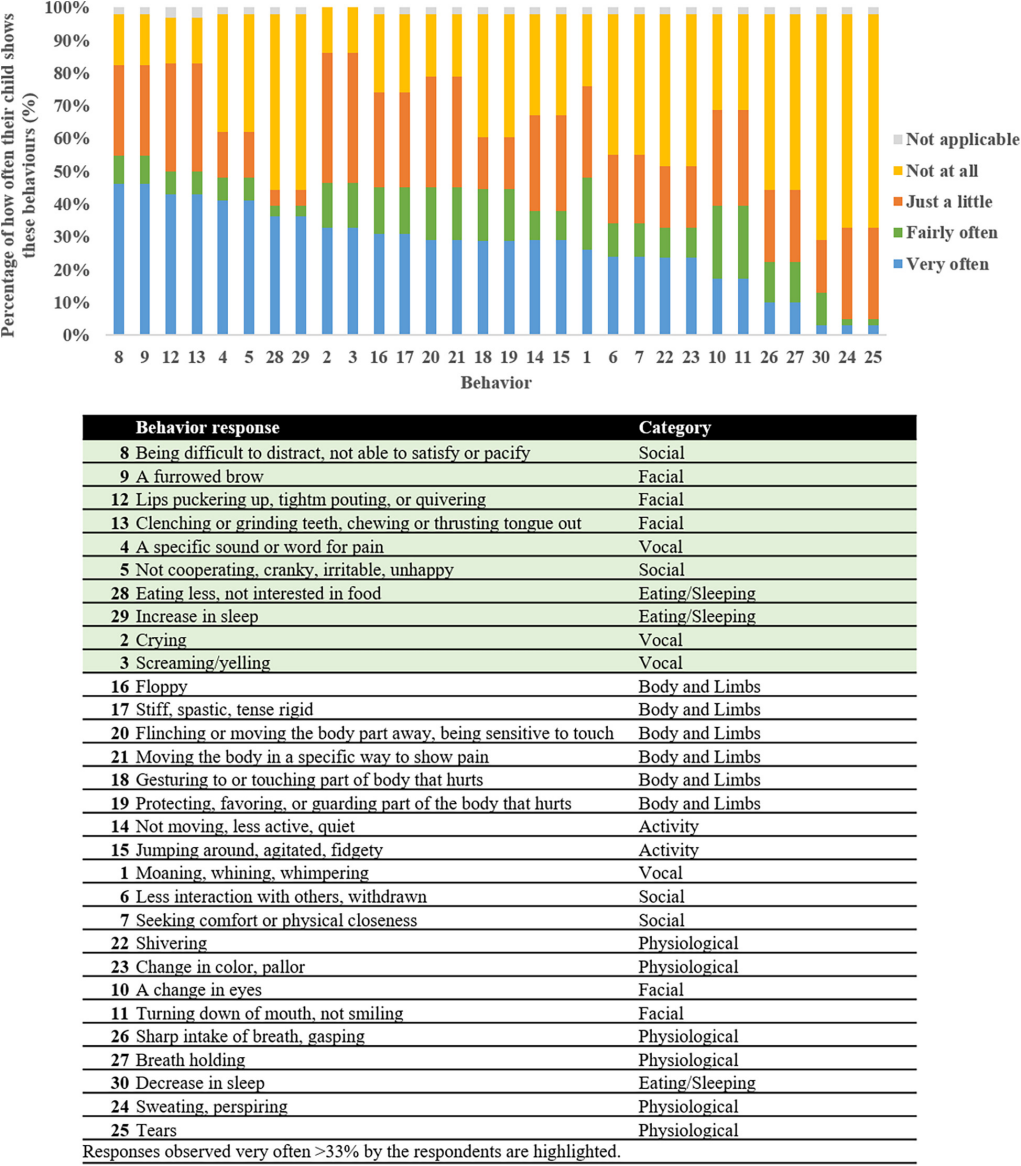
Rate types for each item of the non-communicative Children's pain checklist—revised by the respondents for all painful situations experienced ordered according to frequency of item occurrence. The highlighted responses represent behaviors reported by at least 33% of the respondents.

Nine respondents completed the Non-Communicative Children's Pain Checklist—Revised (Table 4). There were nine painful situations that were reported that the children have not experienced. The situations in which a majority (>50%) of the respondents reported their child has been in included: tripping on stairs (*n* = 6, 67%), stubbing toes (*n* = 6, 67%), hitting into sharp furniture corners (*n* = 5, 56%), and infections (*n* = 6, 67%). When experiencing these situations, 67%–100% (*n* = 4–6) of those respondents reported scores indicating that the child is experiencing pain (total score ≥7) that needs to be addressed. When compiling all the responses reported by the respondents for all painful situations experienced, at least 33% of the respondents reported observing very often vocal, social, facial, and eating/sleeping behaviors (Figure 1).

**Table 4:** Responses for the non-communicative children's pain checklist—revised for different painful situations, *N* = 9.

Painful situations	No	Yes	Total score, Median [Range]	Experiencing pain (Total score ≥ 7)
I. Front entrance				
1. Tripping on stairs	3 (33)	6 (67)	26.5 [3–72]	5 (83)
2. Slipping on ice	8 (89)	1 (11)	74	1 (100)
3. Hit head on door knob	6 (67)	3 (33)	29 [5–31]	2 (67)
II. Living room/bathroom				
4. Stubbing toes	3 (33)	6 (67)	28.5 [1–66]	4 (67)
5. Finger in electric socket	9 (100)	0	NA	NA
6. Hitting into sharp furniture corners	4 (44)	5 (56)	29 [5–57]	4 (80)
7. Falling off furniture	7 (78)	2 (22)	19.5 [4–35]	1 (50)
8. Slipping in tub/shower	5 (56)	4 (44)	26 [8–65]	4 (100)
III. Kitchen				
9. Touching hot, burning items (stove, drinks)	7 (78)	2 (22)	34 [0–68]	1 (50)
10. Biting on tongue or inside the mouth	6 (67)	3 (33)	21 [17–68]	3 (100)
11. Burning of tongue from hot food or frozen food	9 (100)	0	NA	NA
12. Fingers caught in cupboard	5 (56)	4 (44)	28.5 [5–69]	3 (75)
13. Getting cut from sharp objects (knives, utensils, broken glass)	9 (100)	0	NA	NA
IV. Physical				
14. Sore after intense workouts	8 (89)	1 (11)	64	1 (100)
15. Infections (ear, cuts)	3 (33)	6 (67)	30 [17–51]	6 (100)
16. Sore throat, flu	6 (67)	3 (33)	27 [10–41]	3 (100)
17. Growing pains	8 (89)	1 (11)	66	1 (100)
18. Menstrual cramps	9 (100)	0	NA	NA
19. Constipation, upset stomach for different reasons	6 (67)	3 (33)	27 [10–40]	3 (100)
20. Cutting nails too short, into skin	8 (89)	1 (11)	70	1 (100)
V. Traveling				
21. Getting caught in the wheels of wheelchair or between other objects	9 (100)	0	NA	NA
22. Legs falling asleep in wheelchair (bee sting or ants crawling sensations)	9 (100)	0	NA	NA
23. Legs or breathing cramps	9 (100)	0	NA	NA
24. Back pain	9 (100)	0	NA	NA
25. Uncomfortable clothes (prickly wool, too tight)	6 (67)	3 (33)	33 [0–70]	2 (67)
VI. Emotional				
26. Break-ups or loss of loved one	8 (89)	1 (11)	19	1 (100)
27. Being bullied	9 (100)	0	NA	NA
28. Breaking something they like (sentimental object)	6 (67)	3 (33)	36 [30–73]	3 (100)

Data presented as *N* (%) unless otherwise specified. NA, not available, because none of the respondents reported their child experiencing the specific situation.

**Figure 2:**
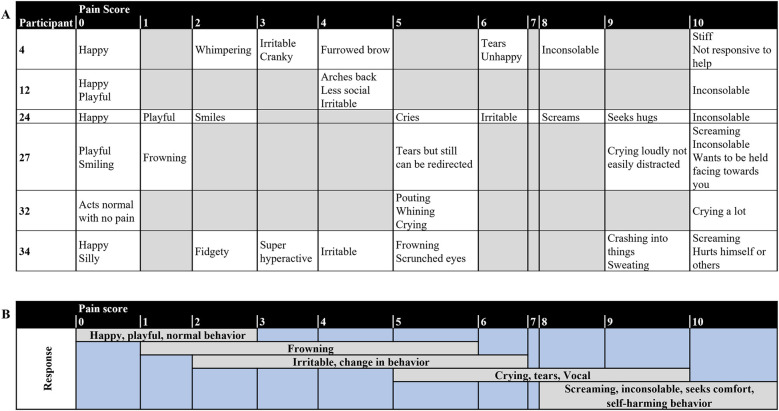
**(A)** Individual and **(B)** summary of responses from the Individualized numeric rating scale of the respondents (*N* = 6).

Six respondents completed the Individualized Numeric Rating (Figure 2A). Upon summative qualitative content analysis (Figure 2B), respondents reported their child to be happy, and present normal behavior during no or mild pain. However, frowning and a change in normal behavior may be observed by the respondents upon mild to moderate pain. During moderate to severe pain, respondents report observing vocal behavior representing pain, inconsolability, and self-harming behavior.

## Discussion

This is the first study to investigate the experience and perception of pain of individuals with Snijders Blok-Campeau Syndrome. We report in 15 individuals with a diagnosis of SNIBCPS, that their caregivers report early and past painful experiences, and have a range of behaviors to express their pain.

At least 50% of the respondents reported past pain experiences as an infant, surgical experiences, illnesses and injuries in their child. However, when a painful experience was reported, respondents' observation of normal or reduced pain responses were nearly 50% split. One caregiver reported recurrent/current pain of their child as very severe for hypermobility and brushing teeth. Studies have shown that untreated or undertreated pain at infancy can lead to increased pain sensitivity (22, 23) and neurodevelopmental and socioeconomic problems (13, 24), and poor pain management or chronic pain in childhood can have consequential effects into adulthood (13, 25–28). Therefore, our findings highlights the need for proper assessment and treatment of pain in children with intellectual disability or developmental delay, especially when “reduced” pain responses are observed. The absence of pain responses in this population may not necessarily mean they feel less pain.

Our findings from the Pain Sensory Questionnaire highlight that although individuals with SNIBCPS may have cognitive impairments, a majority of them are observed to display normal or heightened reactions to diverse sensations. Coursimault et al. reported on a case diagnosed with severe intellectual disability, but associated with a frank happy demeanor, highlighting hypersociability may constitute a suggestive feature of *CHD3* mutations (29). Reduced sensitivity may only be observed in a minority of individuals with SNIBCPS. Nevertheless, this is an important observation to note for caregivers, especially when respondents noted reduced sensitivity to noxious stimuli (e.g., pressure and hard impact), as unreported, undertreated or untreated pain could lead to decreased quality of life (13). Future directions may involve using quantitative sensory testing in this population to determine their somatosensory function and its association with the caregivers' observations. Although quantitative sensory testing primarily relies on the self-report of the subject regarding their sensation of mechanical or thermal stimuli, advances have been made investigating modified quantitative sensory testing protocols for individuals with intellectual or developmental disability (30, 31). Gunderson et al. recently investigated the feasibility of a modified quantitative sensory testing protocol in children with intellectual and developmental disabilities and reported that the modified approach was able to measure tactile reactivity for children with complex communication needs (32). Moreover, due to the rarity of SNIBCPS, proxy-administered quantitative sensory testing with a mobile tool-kit may be a direction to explore (33). A proxy-administered modified quantitative sensory testing approach may be useful to understand the sensory function of individuals with SNIBCPS in relation to developmental and behavioral responses to pain.

Regarding how individuals express pain during painful situations, respondents of our survey report primarily very often observing vocal, social, facial, and eating/sleeping behaviors. These responses were also noted in the individualized numerical rating scales from the respondents. Data from the previously published cohorts of individuals have suggested that most *CHD3* pathogenic mutations were associated with delayed milestones in speech and language, with expressive language being more affected than receptive language (1–3). Moreover, CHD3 protein interacts with FOXP2, which is known to be implicated in language problems in Childhood Apraxia of Speech (34–36). The findings from the INRS highlight the need for proper assessment of individuals with SNIBCPS, especially since vocal expressions were assigned to moderate-to-severe pain scores. The social and eating/sleeping behavioral responses are important to note in this population as they are indirect behaviors related to pain. Findings from the INRS showed that a change in behavior was primarily assigned to mild pain, with inconsolability and self-harming behaviors assigned to severe pain. The INRS has been shown to have good reliability between parents, bedside nurse and research nurse in nonverbal children with intellectual disability (20). Therefore INRS may be a beneficial tool for clinicians, especially in acute or inpatient settings, or when atypical behaviors are reported (37). Healthcare providers for individuals with SNIBCPS should support their families/caregivers to develop their sense of knowledge and skills and to gain confidence in pain assessment (38). Another tool that can be beneficial is the revised and individualized Face Legs Activity Cry and Consolability (FLACC) behavioral pain assessment tool which has been validated in children with cognitive impairment (39). These tools can be used by caregivers to advocate for any under- or untreated pain.

Managing pain in patients with SNIBCPS requires healthcare providers to acquire the knowledge, empathy and compassion to identify and provide proper care. First and foremost, showing empathy and compassion is important to create a safe environment for the patient to feel empowered to express any discomfort non-verbally. Second, a comprehensive assessment of past pain experiences and behaviors observed from the patient's caregiver is important understand the patient's response to painful situations. Finally, an INRS or revised and individualized FLACC scale can be created in collaboration with the patient's caregivers for healthcare providers to effectively identify pain and provide personalized care.

Certain limitations should be considered when interpreting the results of this study. First, this was an anonymous survey with a small sample size with a majority of the participants with SNIBCPS being male and White. According to previous findings (3, 5), a majority of the variants in individuals with SNIBCPS that were inherited were maternally inherited. There was thus a female predominance observed among heterozygote parents, potentially indicating a female protective effect for CHD3 variation. Recent findings have observed no sex bias in the affected probands, or in the severity of the intellectual disability in the novo or inherited cases (3, 5). Nevertheless, our findings cannot be generalized to all individuals with SNIBCPS. With about 170 known cases, future studies with qualitative interviews with families may provide more insight on their experience and response of pain in other demographic groups. Second, many pain-related factors were not included in the data collection of this study, such as comorbidities, medication use, degree of intellectual disability, etc. A comprehensive biopsychosocial pain assessment is therefore warranted in this population. Third, although the parents/caregivers are considered “experts” regarding the pain expression of individuals with SNIBCPS, they can not fully understand their personal pain experience. Pain, as defined by the International Association for the Study of Pain, is “an unpleasant sensory and emotional experience associated with, or resembling that associated with, actual or potential tissue damage” (40). Although there has been work to identify pain biomarkers, there is still headway to bring these biomarkers into point-of care (41, 42). Lastly, the genetic variants involved for each individual was not collected, therefore, it is difficult to determine whether there is a genotype-phenotype relationship regarding the anecdotal decreased pain sensitivity observed in a subset of individuals with SNIBCPS. A registry of individuals with SNIBCPS is currently under development. Nevertheless, a follow-up study using similar methods including genetic information is warranted once the registry is operating.

In conclusion, our study brings awareness about the proper examination of individuals with Snijders Blok-Campeau Syndrome. Despite the small sample size, our findings suggest that pain and injuries may go unreported in individuals with SNIBCPS, and individualized parental observational scales may be beneficial for their healthcare providers. Future studies investigating the relationship between the genotype and the phenotype of this population, especially those with suspected hyposensitivity, is warranted.

## Data Availability

The raw data supporting the conclusions of this article will be made available by the authors, without undue reservation.
